# Authors’s reply

**Published:** 2010

**Authors:** Chamindie Punyadeera, E Marion Schneider, Dave Schaffer, Hsin-Yun Hsu, Thomas O Joos, Fabian Kriebel, Manfred Weiss, Wim F J Verhaegh

**Affiliations:** Department of Molecular Diagnostics, Philips Research, High Tech Campus 12a, 5656 AE Eindhoven, The Netherlands; 1 Experimental Anesthesiology, University Hospital Ulm, Steinhoevelstr. 9, 89075 Ulm, Germany; 2 Philips Research North America, 345 Scarborough Road, Briarcliff Manor, NY 10510, USA; 3 Natural and Medical Sciences Institute, University of Tuebingen, Markwiesenstr. 55, 72770 Reutlingen, Germany; 4 Clinical Anesthesiology, University Hospital Ulm, Steinhoevelstr. 9, 89075 Ulm, Germany

Sir,

We are grateful for your interest in our manuscript and very much appreciate the discussion on biomarkers in the field of sepsis and intensive care patients.[1] Questioning the relevance of biomarkers alongside clinical scores will pave the way for better treatment. You are right in first stressing inhomogeneities in scoring issues as recently pointed out by our group as well.[2]

Nevertheless, SIRS and the sepsis continuum cannot be easily categorized on an individual patient basis. Therefore, it is important to monitor cytokines, soluble receptors and other molecules that are firstly related to pro- and antiinflammatory aspects and to response molecules that might unequivocally distinguish pathogen-associated molecular pattern (PAMP) vs. danger-associated molecular pattern (DAMP).[3]

Besides the functional relevance of biomarkers, practical aspects are of high importance. A biomarker should

1. Respond most rapidly to a given physiological condition or a relevant physiological change
2. A biomarker should be robust and fairly stable
3. A biomarker should be a member of a profile supporting the identification of a biological response in the individual organism

Indeed, our current study is hampered by the fact that the patients’ cohort is small. However, we still provide evidence for the presence of inflammatory markers that indicate a specific biological process. This process is most likely related to the development of immune anergy, i.e. non-responsiveness, because the proteolytic nature of the biomarker spectrum indicates the loss of plasma membrane-associated receptors. Different pathogens causing sepsis may eventually lead to an identical inflammatory biomarker profile. When searching for a clinically relevant biomarker pattern, it is of importance to identify the signaling pathway associated with the clinical condition. The work you referred to represent excellent examples of biomarker studies directing to the importance of other pathways[4] and further stressing the importance of clinical staging.[5] The results of the analysis are, similar to ours, relevant for “evidence based medicine” approaches. A proposal for a future strategy could be to firstly combine the biomarker experiences in a joint effort and secondly, to follow the individual patient’s disease courses in addition to cohort-based findings. Such an effort should prove the relevance of defined biomarker(s) in individualized medicine. In the end, a joint effort using biomarkers of different pathways may be important to select those that may be worthy to be integrated into the currently applied clinical staging. In the predisposition, infection, response, organ dysfunction (PIRO) approach,[6] biomarkers such as IL-6 as well as genetic predispositions given as single nucleotide polymorphisms have been coined to be elementary for clinical scoring as well. However, when biomarkers are included in clinical staging, one has to be careful so as to not increase the application of redundant markers.[7]

Back to the individual patient, we agree with you in that the primary aim should be to diagnose the stimulus leading to the inflammatory response. As stated above, we need to distinguish danger signaling from pathogen-induced signaling responses on the molecular level in order to specifically address the options of an antiinflammatory vs. infection-specific treatment. We therefore feel that the successful distinction of a DAMP vs. a PAMP signaling response is a key element as a future criterion for biomarker selection. Because DAMP primarily activates the inflammasome, all members of the IL-1 family are involved.[8] However, the determination of IL-1ß itself requires a highly sensitive assay as plasma and serum are the most likely candidate biological fluids to be tested and the inflammasome responding cells are most frequently located in tissues and organs. Finally, the IL-1R signaling pathway is in crosstalk with toll-like-receptor specific signaling, being activated by pathogen-derived molecules.[9] Therefore, we need to screen for significant changes in individual biomarkers and, consecutively, provide smart multiparametric calculations for the identification of either one or even multiple pathogenic processes that verify a clinical condition. We thank the authors commenting our publication for their contribution and look forward to further discussions in the field.

## References

[1] Wiwanitkit V (2010). Biomarker for differentiating between SIRS and sepsis: A comment. J Emerg Trauma Shock.

[2] Weiss M, Huber-Lang M, Taenzer M, Traeger K, Altherr J, Kron M, Hay B, Schneider M (2009). Different patient case mix by applying the 2003 SCCM/ESICM/ACCP/ATS/SIS sepsis definitions instead of the 1992 ACCP/SCCM sepsis definitions in surgical patienst: a retrospective observational study. BMC Medical Informatics and Decision Making.

[3] Punyadeera C, Schneider EM, Schaffer D, Hsu HY, Joos TO, Kriebel F (2010). A biomarker panel to discriminate between systemic inflammatory response syndrome and sepsis and sepsis severity. J Emerg Trauma Shock.

[4] Ventetuolo CE, Levy MM (2008). Biomarkers: diagnosis and risk assessment in sepsis. Clin Chest Med.

[5] Herzum I, Renz H (2008). Inflammatory markers in SIRS, sepsis and septic shock. Curr Med Chem.

[6] Levy MM, Fink MP, Marshall JC, Abraham E, Angus D, Cook D (2003). 2001 SCCM/ESICM/ACCP/ATS/SIS International Sepsis Definitions Conference. Intensive Care Med.

[7] Marshall JC (2006). Biomarkers of sepsis. Curr Infect Dis Rep.

[8] Dinarello CA (2009). Immunological and inflammatory functions of the interleukin-1 family. Annu Rev Immunol.

[9] Barksby HE, Lea SR, Preshaw PM, Taylor JJ (2007). The expanding family of interleukin-1 cytokines and their role in destructive inflammatory disorders. Clin Exp Immunol.

